# Relative contribution of neutral and deterministic processes in shaping fruit‐feeding butterfly assemblages in Afrotropical forests

**DOI:** 10.1002/ece3.3618

**Published:** 2017-11-28

**Authors:** Kwaku Aduse‐Poku, Freerk Molleman, William Oduro, Samuel K. Oppong, David J. Lohman, Rampal S. Etienne

**Affiliations:** ^1^ Biology Department City College of New York City University of New York New York NY USA; ^2^ School of Natural Resources University of Energy and Natural Resources Sunyani Ghana; ^3^ Groningen Institute for Evolutionary Life Sciences University of Groningen Groningen The Netherlands; ^4^ Centre for Research and Education in Ecology and Evolution Indian Institute of Science Education and Research Thiruvananthapuram (IISER‐TVM) Thiruvananthapuram Kerala India; ^5^ Department of Systematic Zoology. Ecology Institute of Environmental Biology Faculty of Biology A. Mickiewicz University Poznań Poland; ^6^ FRNR‐College of Agricultural and Renewable Natural Resources Kwame Nkrumah University of Science & Technology Kumasi Ghana; ^7^ Biology Ph.D. Program, Graduate Center City University of New York New York NY USA; ^8^ Entomology Section National Museum of the Philippines Manila Philippines

**Keywords:** Africa, biodiversity, canopy, dispersal, neutral theory, species abundance

## Abstract

The unified neutral theory of biodiversity and biogeography has gained the status of a quantitative null model for explaining patterns in ecological (meta)communities. The theory assumes that individuals of trophically similar species are functionally equivalent. We empirically evaluate the relative contribution of neutral and deterministic processes in shaping fruit‐feeding butterfly assemblages in three tropical forests in Africa, using both direct (confronting the neutral model with species abundance data) and indirect approaches (testing the predictions of neutral theory using data other than species abundance distributions). Abundance data were obtained by sampling butterflies using banana baited traps set at the forest canopy and understorey strata. Our results indicate a clear consistency in the kind of species or species groups observed at either the canopy or understorey in the three studied communities. Furthermore, we found significant correlation between some flight‐related morphological traits and species abundance at the forest canopy, but not at the understorey. Neutral theory's contribution to explaining our data lies largely in identifying dispersal limitation as a key process regulating fruit‐feeding butterfly community structure. Our study illustrates that using species abundance data alone in evaluating neutral theory can be informative, but is insufficient. Species‐level information such as habitat preference, host plants, geographical distribution, and phylogeny is essential in elucidating the processes that regulate biodiversity community structures and patterns.

## 1 INTRODUCTION

A key challenge of community ecology is understanding the link between pattern (e.g., species abundance distributions and species turnover in space) and process (e.g., dispersal and competition). This issue has long fascinated ecologists and remains open even today (Chesson, 2000; Gaston & Chown, 2005; Hubbell, 2001, 2006; Krebs, 2009; McGill et al., 2007; Tokeshi, 1990). Two main but contrasting approaches have been used to explain observed community patterns: deterministic (niche) and stochastic (neutral). The deterministic adaptive niche apportionment hypothesis explains the observed biodiversity patterns as the end products of interspecific interactions, particularly competition, and niche differentiation of coexisting species amidst resource diversity (Chesson, 2000; Hutchinson, 1959; Tilman, 1999). Many studies have indeed demonstrated that species differ in their life‐history traits (e.g., Chown & Nicolson, 2004; Mazer, 1989), and that competition is commonly observed among species in nature (Tilman, 1994, 1999). However, the key question remains: How much do these differences contribute to determining community structure?

The alternative is that communities are unstructured collections of species that have happened to be adapted to the same biome. This neutral theory of biodiversity (Caswell, 1976; Etienne & Alonso, 2007; Hubbell, 2001) is a generalization of the dynamic equilibrium island biogeography model presented by MacArthur and Wilson (1967). This theory emphasizes dispersal limitation as the key process that fashions beta diversity (species turnover in space) as well as species abundance distributions. Neutral theory assumes that all trophically similar species are functionally equivalent. This assumption clearly challenges the classical niche apportionment held by ecologists for decades. Nevertheless, the neutral model has been demonstrated to fit empirical data rather well (e.g., Condit et al., 2002; Latimer, Silander, & Cowling, 2005; Perry, Enright, Miller, Lamont, & Etienne, 2009), and in some cases better than all other relative species abundance models (Volkov, Banavar, Hubbell, & Maritan, 2003) but see McGill (2003). Consequently, the neutral theory has gained status as the quantitative null model for ecological community structure (Alonso, Etienne, & McKane, 2006; Ellwood, Manica, & Foster, 2009; Leibold & McPeek, 2006; Wennekes, Rosindell, & Etienne, 2012) but see (McGill, Maurer, & Weiser, 2006). The question therefore is: What is the relative importance of neutral and deterministic processes in shaping community structure?

Neutral theory in its simplest, spatially implicit, form models population dynamics at two community levels (hierarchical model): a local community and a metacommunity. The local community consists of an assemblage of trophically similar species that (potentially) compete for the same or similar resources in a localized area and is connected to the larger regional pool of species (metacommunity) through dispersal. The metacommunity is maintained by the balance between speciation and extinction. Stochastic ecological processes of birth, death, and immigration are assumed to operate at the local community level. The neutral model requires just two parameters to characterize an ecological community. One parameter is the fundamental biodiversity number θ, which summarizes the speciation process in the metacommunity and is a function of both the metacommunity size (*J*_M_) and the rate (*v*) at which new species arise at random when an individual mutates to become a new species, a process assumed to be similar to mutation of alleles in genetics. The other fundamental parameter is the migration parameter *m* (or equivalently fundamental immigration number *I*; Etienne & Alonso, 2005) which measures the probability of migration or dispersal from the metacommunity into a local community when an individual leaves the local community via death (Hubbell, 2006; Etienne, Alonso, & McKane, 2007; Etienne, 2009a, 2009b). Low *I* values suggest either high dispersal limitation or high establishment limitation or both.

Nearly all evidence in support of neutral theory is restricted to sessile (space‐limited) species (Condit et al., 2002; Hubbell, 2001; Latimer et al., 2005; Perry et al., 2009). Compared to mobile organisms, sessile species generally lack the luxury of deciding where they and their offspring should occur in an ecological system, making lottery effects of establishment more plausible. To fully appreciate the strengths and weaknesses of neutral theory as a universal model, we must as well evaluate the model and its predictions in more mobile organisms. Here we will focus on butterfly communities. Butterflies are by far the best known and most studied larger group of organisms, apart from plants and vertebrates. Both ecological and evolutionary information such as species abundance distributions, species’ traits (e.g., habitat preferences and host plants), geographical distribution, and phylogenies are available for many butterfly species. This information can be used to test to what extent species traits that may be related to deterministic community assembly regulate community patterns.

Many studies attempting to evaluate neutral theory empirically have followed three standard steps (e.g., Condit et al., 2002; Hubbell, 2001; Latimer et al., 2005; Perry et al., 2009; Volkov, Banavar, Hubbell, & Maritan, 2007). First, they estimated the key model parameters (θ, and *m* or *I*) from samples of the species abundances. Then, they used the estimated parameters values to generate artificial communities. Finally, the actual test of neutral theory involved the comparison of the predicted ecological patterns or communities with those of the real biological surveys. However, this approach should be regarded as a preliminary step of evaluating a model (McGill et al., 2006, 2007), because many theories can produce similar patterns of species abundances (Du, Zhou, & Etienne, 2011; Haegeman & Etienne, 2011).

Another approach to evaluating neutral theory is to test the assumptions or predictions of the theory empirically using both species‐specific data and information other than species abundance distributions (McGill et al., 2006). For instance, neutral theory assumes that species traits have no impact on local community structure. The theory asserts that abundance in a local community is determined entirely by ecological drift and in the strict interpretation of neutrality species‐level traits such as habitat preferences, physiological tolerances, and dispersal abilities should not correlate with abundance in a local community. These are predictions that can be evaluated in butterfly assemblages using an extrinsic dataset. In particular, fruit‐feeding butterfly communities tend to show clear niche segregation in the form of vertical stratification (species occurring mainly in the understorey or in the canopy; DeVries, 1988).

Here, we explore the community structure of fruit‐feeding butterflies using species abundance data from two relatively proximal Afrotropical forest communities in Ghana and one remote community in Uganda for which we have reliable abundance data for both canopy and understorey. Specifically, we (1) fitted the standard neutral model simultaneously to multiple samples of butterfly abundances at local and regional scales, (2) tested the within‐species consistency of vertical stratification across the three forests, and (3) assessed the extent to which species‐specific morphological traits and geographical range size (as proxies for dispersal) predicts its occurrence or relative abundance in particular sites or strata.

## 2 METHODS

### 2.1 Study sites

The study was conducted in two protected forests in Ghana (Bia National Park and Bobiri Forest Reserve) and one in Uganda (Kibale National Park). Bia National Park (BIA) is found in the southwestern part of Ghana and borders the forests of Côte d'Ivoire to the west. BIA (06°20′N 06°39′W) covers a total area of 304 km^2^, and lies in a transitional zone between moist semi‐deciduous and moist evergreen zone and forms part of the upper Guinea rainforest—one of the Conservation International global biodiversity hotspots (Myers, Mittermeier, Mittermeier, da Fonseca, & Kent, 2000). Bobiri Forest Reserve (BOB) is located in the middle belt of Ghana (~200 km from BIA) and lies within the moist semi‐deciduous forest zone. BOB (6°25′N 2°40′W) covers about 50 km^2^ and is mainly managed for timber production. A similar fruit‐feeding butterfly dataset (Molleman, Kop, Brakefield, De Vries, & Zwaan, 2006) from Kibale National Park (KIB) was used to compare the results, and neutral theory model parameter values as the theoretical metacommunity is extended from “Ghana” to “Africa.” KIB (0°35′N 20°39′W) is located in western Uganda and at least 3,500 km from BOB and BIA. It lies in a transition between lowland rain forest and submontane forest and is generally classified as a moist evergreen forest and covers an area of 560 km^2^.

### 2.2 Butterfly sampling

We sampled butterflies using fruit‐baited traps between August 2006 and June 2007 in BIA and December 2006 to November 2007 in BOB. The sampled butterflies were almost exclusively Nymphalidae, which is a well‐defined clade recovered by molecular phylogenetics (Wahlberg et al., 2009). Our sample pool therefore meets the requirements of Hubbell's neutral ecological community; trophically similar, sympatric species in a local area compete for the same or similar resources and share a common suite of predators (Hubbell, 2001). Furthermore, bait trapping allowed for sampling the different areas with standardized effort.

Nevertheless, bait trapping techniques also have biases (Hughes, Daily, & Ehrlich, 1998). There may be some fruit‐feeding butterflies that are rarely lured into baited traps. Even among those likely to be trapped, some probably would be more strongly attracted than others (Molleman, van Alphen, Brakefield, & Zwaan, 2005), and some are more likely to escape than others; thus, the relative abundances of species caught may not perfectly reflect the relative abundances of fruit‐feeding species in the local community. However, other techniques such as the use of butterfly nets or visual surveys restrict the sampled butterflies to low and slow flying, and conspicuous species groups, which may not necessarily be closely related phylogenetically and trophically similar. In Ghana, traps were baited with mashed bananas mixed with palm wine. Sampling of fruit‐feeding butterflies was performed on transects. Seven (in BIA) and six (BOB) trap stations were established on each transect (four in each local community) at ~100 m intervals. At each trap station, two fruit‐baited traps were installed: one at the forest canopy and the other at the understorey. Canopy traps were suspended between 20 and 30 m above ground level using thin nylon ropes running over branches of emergent trees, such that they could be serviced directly when the nylon ropes were lowered. The understorey traps were set between 0.1 and 0.2 m above the forest floor. Traps were inspected and (re‐)baited continuously every 24 hr for six consecutive days in each month for 1 year. Bait eaten by rodents and other mammals and traps heavily infested with ants were replaced or refreshed on the day of detection. Otherwise, we refreshed all baits every 2 days, using the original stock of bait prepared on the first day.

Some trap stations could not be used at certain times of the sampling period because their canopy traps were either pushed down by falling tree branches, heavy rainstorms or got stuck in the tree canopy branches during sampling. In such cases, abundance data from the corresponding understorey traps were also discarded to correct for sample effort between the two strata. In total, the quantitative sampling protocol described generated a total of 1,974 and 1,812 trap‐days in BIA and BOB, respectively. For details of the experimental setup in KIB, we refer to Molleman et al. (2006), which did not substantially differ from the setup in Ghana. Specimens were identified to species and grouped into respective taxonomic units (putative species groups, genera, subfamilies) following the proposed higher‐level classification for Nymphalidae by (Larsen, 2005).

### 2.3 Estimating neutral model parameters

We first partitioned the species abundance dataset into three, to reflect the three local communities, namely BIA, BOB, and KIB. We then aggregated the data across the three local communities to form (1) one “combined” but not lumped sample (as species’ and local community identities were maintained in the sample) and (2) three samples of pairs of “combined” local communities; that is, BIA & BOB, BIA & KIB, BOB & KIB. We estimated the neutral model parameters (θ and *I*) for each of the four samples using maximum‐likelihood estimation neutral sampling formulae for multiple samples with varying dispersal limitation (Etienne, 2009a, 2009b). Like the original sampling formulae (Etienne, 2005; Etienne et al., 2007), these sampling formulae assume point mutation as the speciation process and model local communities as spatially separated samples (spatially implicit model). Unlike previous frameworks (Etienne, 2005; Etienne et al., 2007), however, Etienne's (2009a) sampling formula allows for estimation of model parameters and their standard deviation even when the samples (in our case local communities) have different degrees of dispersal (recruitment) limitation.

The sampling formula provides an expression of the probability (*p*[*D*|θ,*I*,*J*]) of observing a particular species abundance dataset *D*, given the neutral model parameters (θ, *I*) and the number of individuals in the sample (*J*). We estimated the neutral model parameters using the code provided in Etienne (2009a, 2009b). Using different starting values, we re‐ran the optimization algorithm at least four times for each “combined sample” to increase the likelihood that we found the global (rather than a local) likelihood optimum. For each of the four “combined samples,” we further partitioned the data into canopy and understorey to reflect the two sampled stratum communities and estimated the model parameter values for each stratum community. We evaluated Hubbell's neutral model in fruit‐feeding butterflies at two metacommunity scales: the “Ghana” metacommunity scale (when only BIA and BOB samples were considered) and the “Africa” metacommunity (when all three local communities are considered).

### 2.4 “Exact” test of neutrality

The second stage of the direct model evaluation employed Etienne's (2007, 2009a) “exact” test of neutrality. This is a general test of neutrality that does not require an alternative (usually niche‐based) model for its evaluation. The test simply involves a comparison of the realized configuration with the probabilities of artificial configurations generated using the model parameter estimates (Etienne, 2007). To implement this test, we simulated 100 artificial communities using the model parameters (θ, *I*) and sample size vector of the observed data (*J*). We then computed for the real data and each of the 100 simulated communities the maximum log‐likelihood and the dissimilarity (Bray‐Curtis) between local community pairs.

To assess the extent to which our neutral model generated artificial communities resembling the observed data, we compared the maximum log‐likelihood value of the real dataset to the frequency distribution of the values of the simulated communities. We performed a similar test with the Bray‐Curtis values to assess the extent to which the observed species turnover departs from those expected under neutrality. We would conclude that the observed community is highly unlikely to be structured by neutral processes if the probability of the real data is significantly smaller than most of the artificial datasets (Etienne, 2007). If, however, the observed community structure is similar to the artificial communities, then we cannot reject neutrality as a plausible driver of the observed biodiversity pattern.

### 2.5 Species distributional range

**Figure 1:**
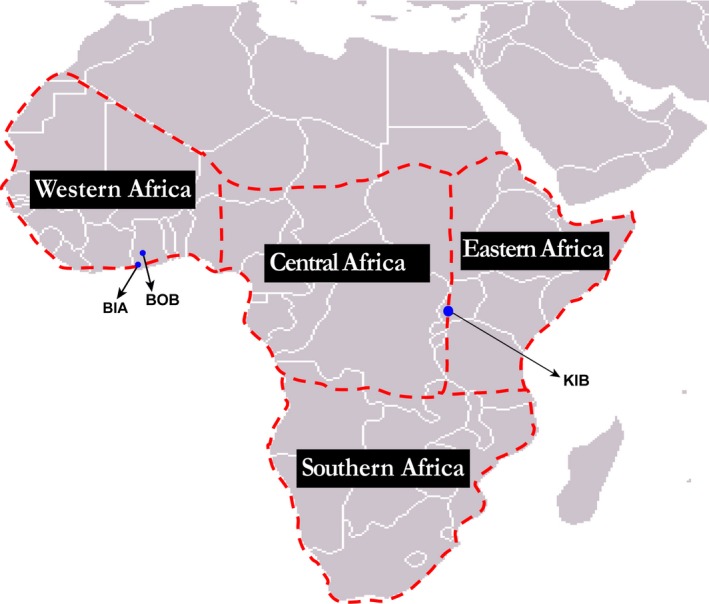
Map of Africa showing the geographical locations of three study areas; BOB (Bobiri Forest Reserve, Ghana), BIA (Bia National Park, Ghana) and KIB (Kibale National Park, Uganda). The dashed lines denote the biogeographical boundaries in Africa based on previous butterfly biogeographic studies (e.g., Carcasson, 1964; Larsen, 2005)

To evaluate the plausibility of the dispersal tendencies impartially, suggested by the neutral model, we obtained species‐specific distributional range information of our sampled species using literature (Larsen, 2005; Williams, 2016). Based on previous biogeographical studies (e.g., Aduse‐Poku, Vingerhoedt, & Wahlberg, 2009; Carcasson, 1964) of Afrotropical butterflies, we partitioned the present distribution of our sampled species into four biogeographical regions; Western African (W), Central Africa (C), Eastern Africa (E), Southern Africa (S) as indicated in Figure 1. We included Madagascar and all surrounding lesser islands as part of southern Africa. The biogeographic distributional range of each sampled species was scored between one and four based on its present distribution on the African continent. A score of one denotes species occurring in only one of the four biogeographical regions in Africa outlined above. A score of four denotes species distributed in all four zoogeographical regions. To correct for geographic size differences, the biogeographic regions were weighted using their approximate land areas. The concomitant weighted scores (as E = 1, S = 1.4, W = 1.2, C = 1.5) were then used as a multiplication factor in computing a *Z*‐score (sized‐corrected zoogeographical score) for each species.

### 2.6 Estimation of recruitment limitation

The recruitment limitation parameter estimates (*I*) of the neutral model inform us about the migration tendencies in the different local communities. Differences in *I* between local communities for instance suggest either that the local communities differ in the ease with which they are reached by dispersal (e.g., hindrance due to the presence of physical barriers) or that they differ in the success of establishment of new arrivals in the local community (Jabot, Etienne, & Chave, 2008). We would expect to find more (individuals of) species with relatively wider distributional range (high dispersal abilities) in communities with high *I*‐values compared with communities with low *I*‐values. To evaluate the plausibility of the migration tendencies impartially, suggested by the neutral model, we obtained species‐specific distributional range information of our sampled species.

### 2.7 Comparing community structure across understory and canopy

We tested the null hypothesis of no difference between the species abundance distribution of the canopy and understorey communities using a two‐sample Kolmogorov–Smirnov test (Magurran, 2004). The Morisita‐Horn index was used to assess similarity in species composition between fruit‐feeding butterfly populations at the canopy and understorey. This index is considered one of the most robust quantitative beta diversity estimators (Magurran, 2004). It quantifies species turnover in terms of both the identities and abundances of species. The index value ranges from 0 (when no species are shared between the compared communities) to 1 (when the compared communities comprise the same species in identical proportions). We also used the classic Sørensen index (Magurran, 2004) to further explore species turnover. Unlike Morisita‐Horn, the Sørensen index (*C*_s_) is simple to calculate and interpret, and based on presence–absence rather than abundance data. All biodiversity indices were computed using the EstimateS software (Colwell, 2009).

### 2.8 Morphometric data and phylogenetic independent contrast

For each sampled individual, using vernier calipers five morphological parameters were measured: (1) Wing length *L*_W_ (forewing base to apex), (2) Wing width *W*_W_ (distance between the leading and trailing edge of the forewing), (3) Thoracic length *L*_T_ (section between the head and abdomen), (4) Thoracic width *W*_T_ (distance between forewing bases), and (5) Abdomen length *L*_A_. Due to high colinearity in morphological traits, we condensed some variables into single factors. For instance, thoracic stoutness (*W*_T_/*L*_T_) was used as a combined effect of thorax length and thorax width. Likewise, wing aspect ratio (4*L*_W_^2^/*W*_W_ ×* L*_W_) indexed the forewing parameters. To correct for sexual size dimorphism, morphological data were taken from male specimens only.

Using a phylogenetic tree reconstructed from a five‐gene matrix of sequences used in previous species‐level phylogenetic studies (Aduse‐Poku, Brakefield, Wahlberg, & Brattström, 2017; Aduse‐Poku et al., 2009, 2016; Monteiro & Pierce, 2001; Van Velzen, Wahlberg, Sosef, & Bakker, 2013), a statistically independent set of contrast values (see Felsenstein, 1985; for details of this method) were computed for each measured morphological trait and squared root‐transformed species abundance. This method, referred to as phylogenetic independent contrast (PIC), removes the inherent phylogenetic signals in the dataset (Felsenstein, 1985). These PIC analyses were performed separately for the canopy and understorey communities and for the lumped community. The PIC analyses were limited to BOB and BIA where DNA sequences and morphological data were available for most taxa. The effects of variation in any of the species‐level morphological traits between species on the overall abundance were performed using regression models. All analyses were implemented in R Development Core Team (2014).

## 3 RESULTS

### 3.1 Overview

A total of 7,556 individuals representing 154 butterfly species were captured from the two locations in Ghana (Table 1). All specimens but three were identified to species level (Electronic Supplementary Material, ESM1). Captured Butterflies came from 32 genera and eight subfamilies; all members of the Nymphalidae family. Table 1 summarizes the abundance, richness, and sampling efforts at each local community. A total of 32,308 individuals belonging to 94 species were trapped in KIB (Molleman et al., 2006). BIA was by far the most species rich (139), despite being the community with the fewest sampled individuals. Species abundances at the understorey were generally higher (fourfold to sixfold) than at the forest canopy. We also recorded more species (range 90–109) at the understorey than at the canopy level (range 54–75).

**Table 1:** Number of individuals and species captured in each local community. Pooled data resulted from lumping of the forest canopy and understorey data. Understorey and canopy denotes that each vertical stratum community data is considered separately. Trap‐days are calculated as the number of traps installed at a locality multiplied by the number of times sampled. One trap‐day is equivalent to one trap sampled for a day (within 24 hr after setting out trap). KIB, BIA and BOB denote Kibale National Forest in Uganda, Bia National Park in Ghana and Bobiri Forest Reserve in Ghana respectively

Summary statistics	Data set								
	Pooled			Understorey			Canopy		
	KIB_POL_	BIA_POL_	BOB_POL_	KIB_UND_	BIA_UND_	BOB_UND_	KIB_CAN_	BIA_CAN_	BOB_CAN_
Abundance	32,310	2,764	4,782	27,960	2,187	4,151	4,350	577	631
Richness	94	139	111	90	109	90	75	59	54
Trap‐days	6,952	1,974	1,812	3,476	987	906	3,476	987	906

### 3.2 Community structure as described by neutral model parameters

We estimated neutral model parameters values (θ and *I*) for different samples from the three local fruit‐feeding butterfly communities (Table 2). The neutral model parameter estimates (θ, *I*) hinted at two kinds of ecological communities, depending on the scale of metacommunity considered. On a “Ghana” metacommunity scale (i.e., when only BIA and BOB samples are considered), the θ and *I* estimates suggested a closed ecological system with low regional diversity (low θ) and low dispersal and/or recruitment limitation (high *I*). In contrast, on the “Africa” metacommunity scale (i.e., “combined” sample of the three local communities), the parameter estimates depicted a system of high regional diversity (high θ) and strong dispersal and/or recruitment limitation (low *I*) (Table 2). This reflects the large spatial separation between Ghana and Uganda.

**Table 2:** Neutral parameter estimates for samples from three local fruit‐feeding butterfly communities (BOB [Bobiri Forest Reserve], BIA [Bia National Park], and KIB [Kibale National Park], using Etienne (2009a, 2009b) sampling formulae for multiple samples with varying degrees of dispersal limitation. *J* and *S* are the number of individuals and species respectively in each local community denoted as BOB, Ghana; BIA, Ghana; KIB, Uganda. *I*_BIA_, *I*_BOB_ and *I*_KIB_ are the recruitment parameter estimates for BIA, BOB and KIB respectively. θ is the fundamental biodiversity number. *p*_MLE_ and *p*_BC_ are the probabilities that the log‐likelihoods and Bray‐Curtis indices of the model simulated communities deviate significantly from the observed community. The values next to the plus and minus sign (±) are the standard deviation of the parameter estimates

Data set	Sample size and species richness		Maximum likelihood parameter estimates					Neutrality test	
	*J*	*S*	θ	*I*_BOB_	*I*_BIA_	*I*_KIB_	Loglik	*p*_MLE_	*p*_BC_
Pooled									
BOB + BIA + KIB	4,782, 2,764, 32,310	111, 139, 94	96.1 ± 10.1	47.3 ± 6.63	97.0 ± 14.8	17.3 ± 2.0	−1,079.4	0.478	–
BOB + BIA	4,782, 2,764	111, 140	49.5 ± 5.31	91.9 ± 13.6	324.6 ± 80.8		−516.5	0.473	0.651
BOB + KIB	4,782, 32,310	111, 94	171.9 ± 27.6	29.4 ± 3.60		16.6 ± 2.0	−572.7	0.536	0.746
BIA + KIB	2,764, 32,310	140, 94	185.0 ± 27.8		51.2 ± 6.30	16.0 ± 1.9	−561.4	0.369	0.229
Understorey									
BOB + BIA + KIB	4,151, 2,187, 27,960	90, 109, 90	101.0 ± 11.8	30.1 ± 4.26	55.5 ± 8.23	16.7 ± 2.0	−909.0	0.638	–
BOB + BIA	4,151, 2,187	90, 109	42.3 ± 5.09	69.7 ± 11.8	212.2 ± 54.3		−424.5	0.936	0.796
BOB + KIB	4,151, 27,960	90, 90	179.1 ± 32.3	21.2 ± 2.71		15.6 ± 1.9	−493.9	0.674	0.770
BIA + KIB	2,187, 27,960	109, 90	200.7 ± 35.1		34.3 ± 4.36	15.1 ± 1.8	−477.9	0.558	0.229
Canopy									
BOB + BIA + KIB	631, 577, 4,351	54, 59, 75	73.9 ± 10.8	28.8 ± 5.90	33.5 ± 6.77	18.3 ± 2.5	−455.9	0.634	–
BOB + BIA	631, 577	54, 59	20.7 ± 3.18	126.2 ± 36.6	293.1 ± 171		−183.0	0.473	0.651
BOB + KIB	631, 4,351	54, 75	142.8 ± 31.5	18.8 ± 3.32		16.8 ± 2.4	−242.5	0.815	0.331
BIA + KIB	577, 4,351	59, 75	147.1 ± 31.5		21.7 ± 3.71	16.6 ± 2.3	−241.4	0.802	0.558

There were considerable and sometimes significant differences in the degree of dispersal/recruitment limitations among the three local communities. Dispersal/recruitment parameter estimates for BIA (*I*_BIA_) were consistently the highest, regardless of the metacommunity scale looked at. *I*_KIB_‐values on the other hand were always the lowest in all its “combined” samples (Table 2). Between BOB and BIA (i.e., within the “Ghana” metacommunity), *I*_BIA_ values were nearly three times higher than *I*_BOB_.

### 3.3 Neutrality test

**Figure 2:**
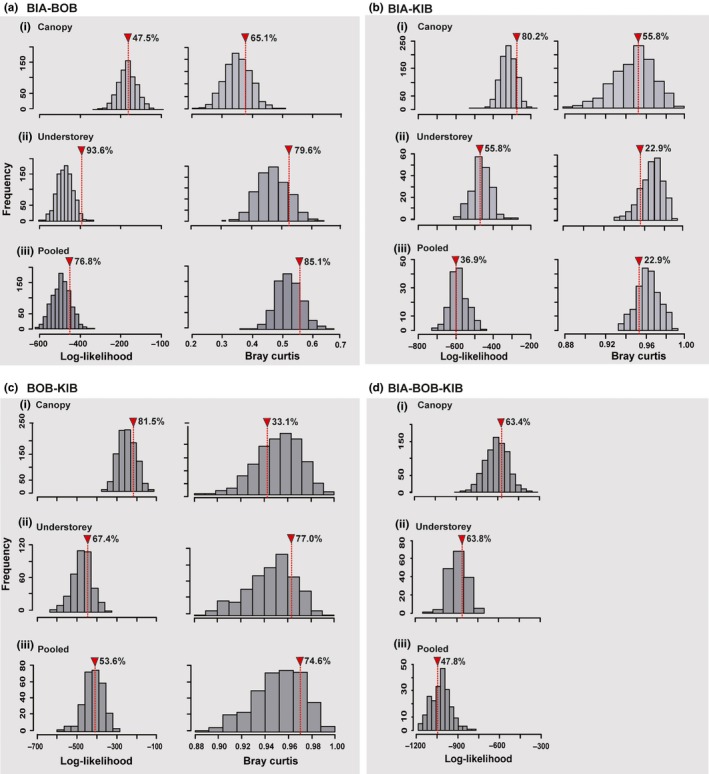
Test of departure from neutrality using the Etienne's (2007, 2009a, 2009b) “exact” test of neutrality formulae. The test involves a comparison of the realized configuration with the probabilities of 1,000 artificial configurations generated using the model parameter estimates (Table 2). The arrow indicates the position of the observed data in relation to the simulated neutral communities. Values besides the arrow show the percentage of simulated communities with values less than the observed. Understorey and Canopy denotes that each vertical stratum community data is considered separately. Pooled is when the forest canopy and understorey data are lumped[pooled]

The “exact” test of neutrality suggested that we cannot reject neutrality and/or dispersal limitation as a plausible explanation for the patterns of abundance distributions in the three fruit‐feeding butterfly communities (Table 2). Indeed, communities simulated by our neutral model tended to resemble the observed data; the observed likelihood was well within the frequency distribution of the simulated likelihoods (Figure 2). This was true when the canopy and understrorey communities were analyzed separately and when they were pooled. Likewise, the observed species turnover (measured with the Bray‐Curtis index) did not depart significantly from those simulated under neutrality at the “Ghana” metacommunity scale. The situation was no different when the metacommunity was extended from “Ghana” to “Africa” to include the samples from KIB.

### 3.4 Evaluating the plausibility of the migration tendencies suggested by the neutral model

**Figure 3:**
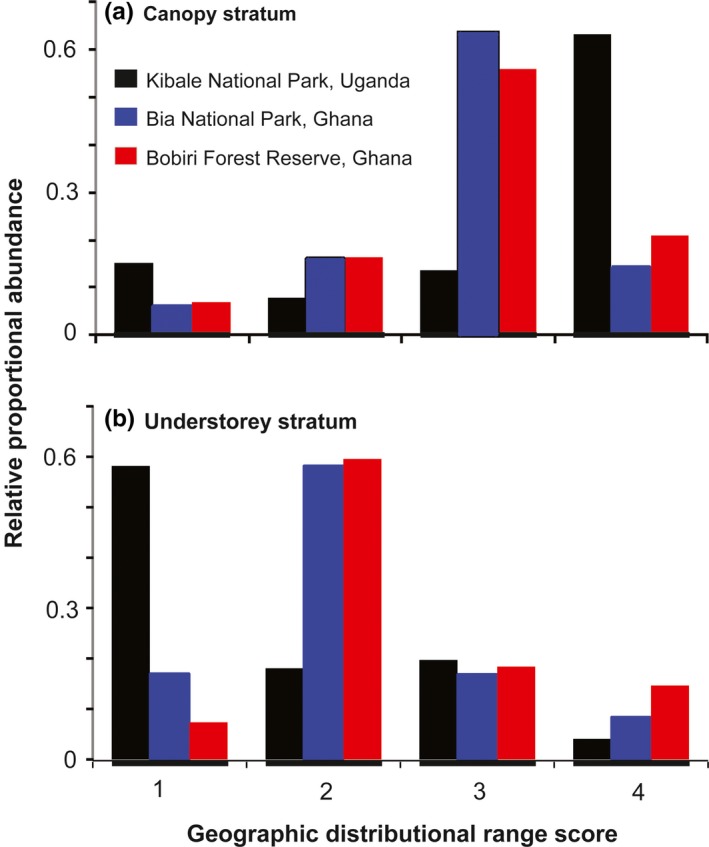
Histogram of the relative proportional abundance of individuals with different distributional ranges

The neutral model parameter estimates (*I*) suggested low dispersal limitation in BIA compared to BOB. The high *I*_BIA_ values suggest that either BIA is relatively easier to reach by dispersal (i.e., less hindrance due to few or no physical barriers) or that it is easier for dispersers to establish themselves in BIA. Within the “Ghana metacommunity,” our neutral model parameters (*I*) also suggested less dispersal and/or recruitment limitation in the canopy compared to the understorey. In other words, it is relatively easier for immigrants to establish in the canopy than in the understorey. To evaluate the plausibility of these model predictions, we compared the abundance of individuals with their different distribution ranges in the canopy and understorey stratum communities. The results of our distributional range analyses were consistent with those expected under neutrality. There were relatively more individuals (~78%) of taxa with higher dispersal abilities (thus, wider *Z*‐score scores; ≥3.5) at the canopy compared to the understorey (Figure 3). Nearly 70% of the individuals trapped at the forest understorey in both BOB and BIA were restricted to one or two of the biogeograpical regions with *Z*‐score ≤2.7 (Figure 3). However, when the metacommunity was extended from “Ghana” to “Africa” to include the dataset from KIB, our neutral model parameter estimates suggested a rather opposite trend, conflicting with the results from the independent distributional range analyses (Figure 3).

### 3.5 Community structure and vertical stratification of fruit‐feeding butterflies

We observed considerable differences in both the structure and compositions of fruit‐feeding butterfly assemblages found at the two strata. On average, there was ~52% overlap in species presence (measured as Sørensen index) and ~11% overlap when relative abundances are taken into account (measured by Morisita‐Horn) between the understorey and canopy communities. The similarity values were relatively lower (about half the average) in Ghana; Sørensen 36%, Morisita‐Horn 9%, compared with KIB Sørensen 84% and Morisita‐Horn 15%. The species abundance distribution patterns in the forest understorey were significantly different from those observed at the canopy. This was true for all the three sampled locations (Kolmogorov–Smirnov test; for KIB, *D *=* *0.1809, *p *=* *0.003; for BIA, *D *=* *0.2513, *p *<* *0.001; and for BOB, *D *=* *0.1809, *p *=* *0.003). When we analyzed the species abundance distributions of canopy and understorey samples separately, we found no significant difference in abundance distribution between the two local communities in Ghana (Understorey, *D *=* *0.0954, *p *=* *0.3246; Canopy, *D *=* *0.0302, *p *=* *1). When either BIA or BOB was contrasted with KIB in the separate stratum analysis, we observed significant differences in the fruit‐feeding butterfly community structures in understorey but not in the canopy (ESM3). Although there were no substantial differences in abundance distribution at the canopy between the three study sites, the two communities in Ghana (BOB and BIA) were more similar to each other in abundance distribution than when either was contrasted with KIB.

**Figure 4:**
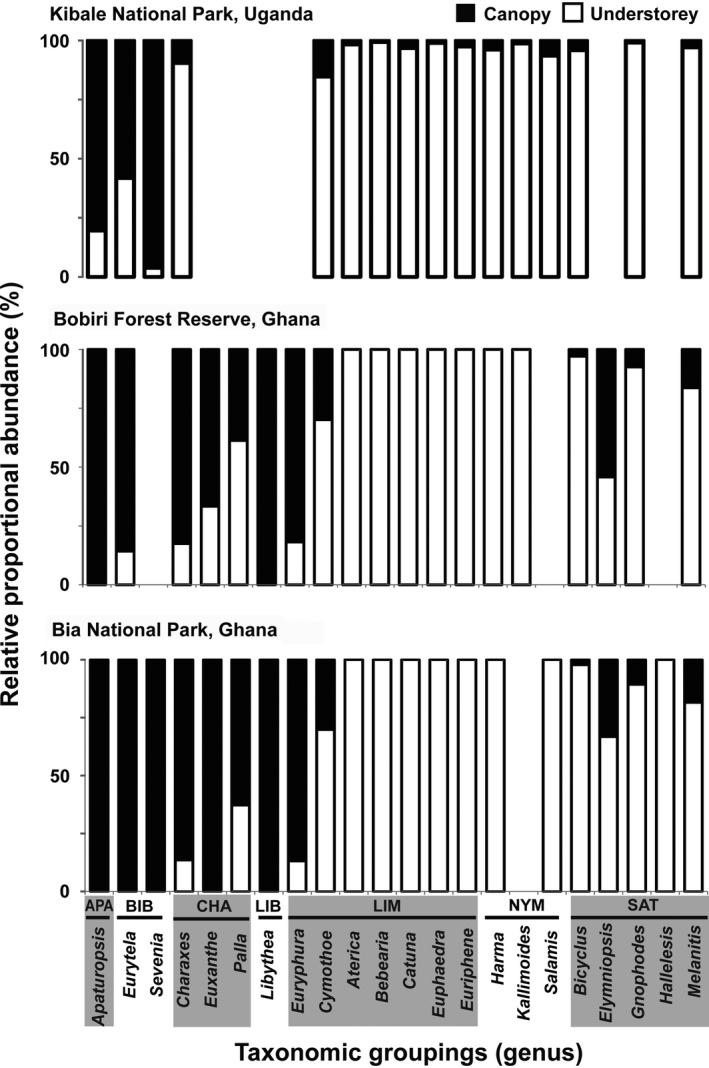
Bar chart of relative percentage proportional abundance of fruit‐feeding butterfly genera and subfamilies at the forest canopy and understory in three protected forests in Africa: Kibale National Park, Uganda, Bobiri Forest Reserve, Ghana, Bia National Park, Ghana. The shortened subfamily names are APA = Apaturinae, BIB = Biblidinae, CHA = Charaxinae, LIB = Libytheinae, LIM = Limenitidinae, NYM = Nymphalinae, SAT = Satyrinae. A gap on the genus axis means that no member of the genus was captured at that particular local community.

The compositions of the butterfly communities were strikingly different between the canopy and understorey at all taxonomic levels: subfamily, genus, and species, and this was consistent across sites (Figure 4, ESM1). Generally, the understorey fruit‐feeding butterfly community was composed mainly of members of the subfamilies Limenitidinae, Nymphalinae, and Satyrinae. The Limenitidinae subfamily is composed of genera such as *Bebearia*,* Catuna*,* Euphaedra*, and *Euriphene* which were predominately captured at the forest understorey. Of the total Limenitidinae individuals sampled in KIB, 7,821 were from the understorey and only 398 (<5%) were captured at the forest canopy. Not even a single of the nearly 2,000 individuals (comprising 56 species) of these four genera was captured at the canopy during the entire sampling period in Ghana. An even more entrenched pattern was exhibited by members of the Satyrinae subfamily. This species‐group contributed the largest (~62%; 21,061 individuals) to the overall understorey species abundance pool and only 4% of the total Satyrinae individuals trapped were recorded from the forest canopy.

In contrast, the canopy was preferred largely by the Charaxinae (*Charaxes* and *Palla*), Apaturinae, Libytheinae, and Biblidinae subfamilies (Figure 4, ESM1). For instance, of the total 958 Charaxinae we trapped in Ghana, an overwhelming 83% were from the canopy (Figure 4). The relative abundances of the *Charaxes* and *Eurytela* species were a bit different among the strata communities in KIB, where the most common *Charaxes* (*C. fulvescens*) is an understory specialist. The Apaturinae subfamily in continental Africa is represented by a single species, *Apaturopsis cleochares*. We recorded 64 individuals of this species in Ghana and all were from the canopy. In KIB, 111 of 138 (84%) individuals of this species were captured in the canopy.

### 3.6 Correlations between morphology and abundance

We found evidence of association between the measured thoracic traits (thoracic width, thoracic length, and stoutness) and species abundance at the canopy in BIA and when BIA and BOB dataset were pooled, but not when BOB dataset was considered alone (Table 3). Aside these, we found no evidence of correlation between the measured morphological traits and species abundance at the understory, canopy, and pooled data.

**Table 3:** Regression of phylogenetic independent contrasted (pic) morphological traits with (log‐transformed) species abundances in the different strata communities in two forests in Ghana. The bold numbers are probability values of 0.05 or less levels of significance of the correlated morphological traits with species abundance

Community	Stratum	Summary statistics	*df*	Wing width	Wing length	Wing index	Thoracic length	Thoracic width	Stoutness	Abdomen length
Bia National Park	Canopy	*F*‐statistic	20	0.665	0.831	0.813	6.374	9.309	6.253	0.176
		Adjusted *R* ^2^	20	−0.016	−0.008	−0.009	0.204	0.284	0.200	−0.041
		*p*‐value	20	0.425	0.373	0.378	**0.020**	**0.006**	**0.021**	0.679
	Understorey	*F*‐statistic	41	0.286	0.538	0.477	0.371	0.311	0.032	0.000
		Adjusted *R* ^2^	41	−0.017	−0.011	−0.013	−0.015	−0.017	−0.024	−0.024
		*p*‐value	41	0.596	0.468	0.494	0.546	0.580	0.860	0.994
Bobiri Forest Reserve	Canopy	*F*‐statistic	19	2.538	0.821	1.202	0.602	0.696	1.162	1.780
		Adjusted *R* ^2^	19	0.071	−0.009	0.010	−0.020	−0.015	0.008	0.038
		*p*‐value	19	0.128	0.376	0.287	0.447	0.415	0.295	0.198
	Understorey	*F*‐statistic	36	0.060	0.168	0.138	0.017	0.099	0.440	0.823
		Adjusted *R* ^2^	36	−0.026	−0.023	−0.024	−0.027	−0.025	−0.015	−0.005
		*p*‐value	36	0.807	0.685	0.712	0.897	0.754	0.511	0.370
Ghana metacommunity (pooled data)	Canopy	*F*‐statistic	24	0.069	0.198	0.163	4.750	5.818	3.783	0.347
		Adjusted *R* ^2^	24	−0.039	−0.033	−0.035	0.130	0.162	0.100	−0.027
		*p*‐value	24	0.794	0.661	0.690	**0.039**	**0.024**	**0.044**	0.562
	Understorey	*F*‐statistic	43	0.119	0.258	0.222	0.016	0.189	0.290	0.547
		Adjusted *R* ^2^	43	−0.020	−0.017	−0.018	−0.023	−0.019	−0.016	−0.010
		*p*‐value	43	0.732	0.614	0.640	0.900	0.666	0.593	0.464

## 4 DISCUSSION

We investigated the relative importance of neutral and deterministic processes in determining fruit‐feeding butterfly communities in Africa. This is one of the first studies testing neutral theory on mobile animals (Jones, Blackburn, & Isaac, 2011). The neutral model fitted our empirical data well with respect to identifying dispersal limitation as one key factor structuring fruit‐feeding butterfly communities. However, we also found evidence for deterministic processes playing a role. Perhaps the most striking property of fruit‐feeding butterfly assemblages is their vertical niche differentiation. The apparent consistency in the kind of species or species groups found at either the canopy or understorey in the three studied communities suggests that fruit‐feeding butterfly assemblages are largely structured by species’ vertical (habitat) preference. If at all, neutral theory should only be applied to understorey or canopy separately. Previous long‐term vertical stratification studies report similar distinct differences in fruit‐feeding butterfly faunal composition between the forest canopy and understorey in Africa (Aduse‐Poku et al., 2012; Fermon, 2002), Asia (Fermon, Waltert, Vane‐Wright, & Muhlenberg, 2005; Schulze, Linsenmair, & Fiedler, 2001) and the Neotropics (DeVries, Walla, & Greeney, 1999; Fordyce & DeVries, 2016).

Potential deterministic processes that could play a role in structuring fruit‐feeding butterfly communities include resource competition during larval and adult stages, and apparent competition via natural enemies. Adult fruit‐feeding butterflies seem to compete for the same resources (e.g., fallen fruits), but there may be some specializations based on proboscis morphology (Molleman, Krenin, et al., 2005). Furthermore, species may differ in competitive ability leading to dominance hierarchies at fruit items (Torres, Osorio‐Beristain, Mariano, & Legal, 2009). During the larval stage, competition among species depends on host‐plant overlap. The vast majority of satyrinaes are thought to be more or less generalistic grass‐feeders as larvae (Larsen, 2005) and could thus potentially compete. In contrast, most Limenitidinae utilize a small number of dicotyledon food plants, mainly growing at or near the forest understorey, and would thus only compete in particular cases. Similarly, the larvae of most of the dominant “canopy species groups” (*Charaxes*,* Palla* and *Apaturopsis*) are usually locally mono‐ or oligophagous tree foliage feeders that would rarely compete with each other (Larsen, 2005). However, given the generally low caterpillar and adult butterfly densities compared to resources (host plants, fallen fruits), it is likely that apparent competition via shared natural enemies may be more important than competition for food, but this has hardly been addressed.

Our “exact” test of neutrality did not lead to rejection of the neutral model. However, failure to reject a neutral model does not necessarily mean that the observed biodiversity pattern is generated by neutral processes alone. A key process structuring community assemblages in neutral theory models is dispersal limitation. Given the geographic distances, it was not surprising that the neutral model detected higher recruitment limitation between the Ghana and Uganda sites than among the Ghana sites. While we cannot exclude that this is a result of habitat filtering (the habitats of the Ghana and Uganda forests selecting for different species based on, for example, climate and host‐plant availability), it is indeed likely that dispersal limitation due to geographic distance plays an important role in determining this general biogeographic pattern.

However, our analyses also provide evidence for dispersal limitation that is intrinsically linked to the life history traits of the fruit‐feeding butterfly species found in the two vertical strata. The measured species‐specific thoracic traits appear to predict species abundance in the canopy community in BOB but not in BIA (Table 3). The lack of statistical support in the latter could be due to the exclusion of key canopy species of BOB from the PIC analyses for unavailability of DNA sequences. The thorax morphology of insects is associated with flight performance and maneuverability (Dudley, 2000; Yokoyama, Senda, Iima, & Hirai, 2013). In general, most canopy species are robust in body structure, have strong flight muscles, and are powerful in flight (Henning, 1989; Larsen, 2005). These species traits enable them to sustain high flights in the canopies for relatively longer periods and also disperse long distances (i.e., make them good dispersers). As a result, isolated forests patches tend to be easier colonized by canopy species than by understory species, because canopy specialists are more mobile (Fordyce & DeVries, 2016) and often comfortable to fly in full sunlight. Therefore, the higher migration tendencies in the canopy community suggested by the neutral model are consistent with the more dispersive morphologies we found in canopy species. This is corroborated by the observation that the canopy species tend to have wider distributional ranges (Figure 3).

However, extending the conceptual metacommunity from “Ghana” to “Africa” by including the KIB samples with those from Ghana (BOB and BIA) in one simultaneous analysis led to conflicting conclusions. For instance, at the “Africa” metacommunity scale, the neutral model parameters estimates suggested less dispersal limitation (although not always significant) at the understorey, compared to the forest canopy. This apparently contradicts the inference at the Ghana level and also the results of the species distributional range analysis which indicates a rather opposite trend; more individuals (of species) with wider distributional ranges at the canopy than at the forest understorey (Figure 3). More so, unlike in the “Ghana” metacommunity, our neutral model parameter (*I* and θ) estimates for the different local communities were frequently not significantly different from each other at the “Africa” metacommunity level (Table 2).

We may explain the weakening in the neutral model parameters estimates’ information as the metacommunity extends from “Ghana” to “Africa” as follows: First, KIB and the two local communities in Ghana (BIA and BOB) may not belong to the same metacommunity. The essence of including KIB in the study was to provide, at least, an approximate answer to a rather unanswered fundamental question; at what distance apart, can two samples or local communities of butterflies (in the current case) be said to belong to the same metacommunity? Etienne (2007) offers a general rule of thumb: for the estimation method to be valid, samples treated as local communities should be separated by distances longer than the typical dispersal of the studied taxa but at the same time, close enough to belong to the same metacommunity. Information on butterfly dispersal distance is currently unavailable but certainly a distance of 3,500 km between KIB and Ghana (BOB and BIA) is intuitively above the typical.

Secondly, it could be that fitting the three communities simultaneously simply results in a poorer fit than for two communities at a time. The log‐likelihood values of the three local communities are approximately double those for two local communities, irrespective of the stratum considered (canopy, understory, or pooled, Table 2). This observation suggests that the same local community data can be adequately fitted by a wide range of values of θ and *I*, either a combination of low θ and high *I*, or vice versa, and this seems to be the case regardless of whether a single global optimum or several local optima exist for the two parameters. Subsequently, when trying to fit two local communities, the parameter fitting flexibility can be stretched sufficiently to allow a combination of a single θ and two *I* values that are still compatible with the data. However, when three communities (in our case BOB, BIA and KIB) are fitted simultaneously, the inherent flexibility of the classical (spatially implicit) neutral theory may no longer suffice.

We have shown that using species abundance data alone in investigating the factors or processes regulating biodiversity community structures and patterns can be informative, but is not sufficient. Neutral theory performed well in identifying dispersal limitation as one key factor structuring fruit‐feeding butterfly communities. By including other useful ecological and evolutionary information, we also identify the vertical dimension of fruit‐feeding butterfly assemblages as an important aspect of community structure. Clearly vertical differences at the scale of meters in fruit‐feeding assemblages are much larger than horizontal ones at the scale of 100s of kilometers. Deterministic dispersal limitation is probably playing a role as some flight‐related morphological traits were found in some cases predictor of species abundance, at least in the canopy stratum. We have shown that among species, differences in the traits of fruit‐feeding butterflies do matter in determining their presence and abundance in ecological communities. These results show that neutral models can be a good starting point to test for the relative importance of deterministic processes and to compare different habitats such as understory and canopy.

## CONFLICT OF INTEREST

None declared.

## AUTHOR CONTRIBUTIONS

KAP and RSE conceived the idea. KAP, WO, and SKO designed the study. KAP and FM collected the field data. KAP and RSE performed the analysis. KAP wrote the manuscript with active participation of WO, SKO, FM, DJL, and RSE.
