# Supplementary figures and images for: Relative contribution of neutral and deterministic processes in shaping fruit‐feeding butterfly assemblages in Afrotropical forests
Source: Ecol Evol. 2017 Nov 28;8(1):296–308. doi: 10.1002/ece3.3618 (PMC5756852; doi:10.1002/ece3.3618)

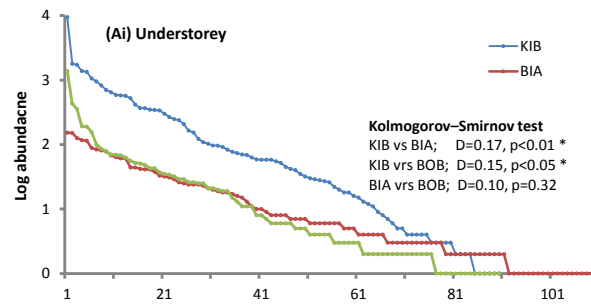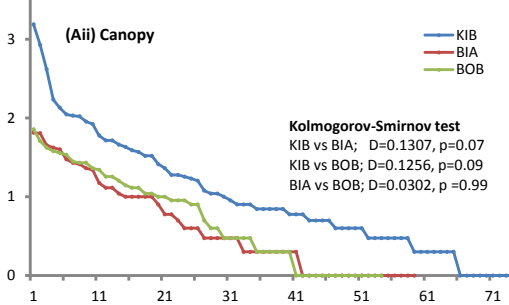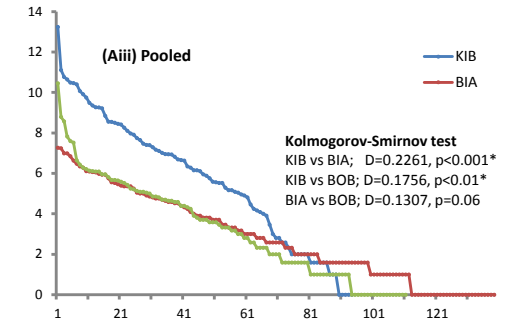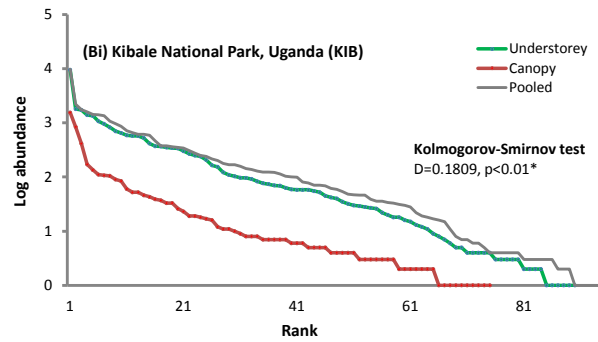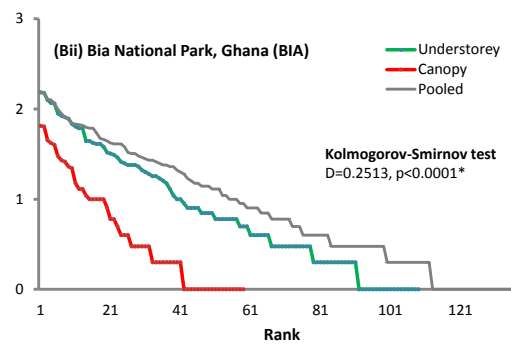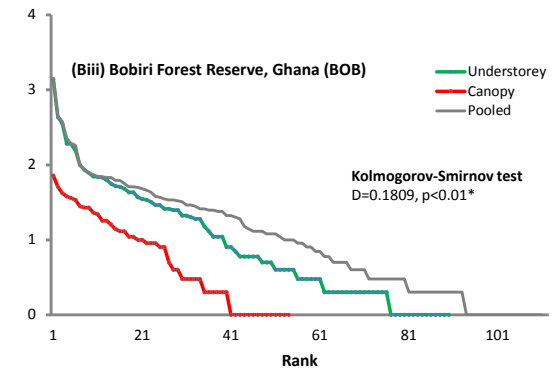

Supplement: Supplementary file 1 [file ECE3-8-296-s001.pdf]
