# Supplementary material for: Relative contribution of neutral and deterministic processes in shaping fruit‐feeding butterfly assemblages in Afrotropical forests
Source: Ecol Evol. 2017 Nov 28;8(1):296–308. doi: 10.1002/ece3.3618 (PMC5756852; doi:10.1002/ece3.3618)
Supplement: Supplementary file 2 [file ECE3-8-296-s002.pdf]

ESM1 Species and relative abundance (abund[a]) of individuals of fruit-feeding butterfly recorded at the understorey (under.) and the canopy strata in each local community.

Subfamily<br>Species	Kibale National Park			Bia Biosphere Reserve			Bobiri Forest Reserve		
	Under.	Canopy	Abund[a]	Under.	Canopy	Abund[a]	Under.	Canopy	Abund[a]
Apaturinae									
Apaturopsis cleochares	27	111	138	--	13	13	--	51	51
Biblidinae									
Ariadne enotrea	55	26	81	--	--	--	--	--	--
Ariadne pagenstecheri	5	1	6	--	--	--	--	--	--
Byblia anvatara	--	--	--	--	--	--	1	--	1
Eurytela dryope	3	3	6	--	1	1	1	3	4
Eurytela hiarbas	590	842	1,432	--	1	1	--	3	3
Neptidopsis ophione	83	39	122	1	--	1	--	1	1
Sevenia boisduvalli	58	1,542	1,600	--	--	--	--	--	--
Sevenia occidentalium	1	46	47	--	2	2	--	--	--
Sevenia umbrina	8	105	113	--	--	--	--	--	--
Charaxinae									
Charaxes ameliae	--	--	--	--	4	4	--	1	1
Charaxes anticlea	3	--	3	--	10	10	1	11	12
Charaxes bipunctatus	77	52	129	1	2	3	2	18	20
Charaxes bocqueti	--	--	--	--	5	5	--	2	2
Charaxes boueti	--	--	--	--	--	--	--	1	1
Charaxes brutus	4	3	7	2	26	28	--	34	34
Charaxes candiope	20	10	30	--	--	--	--	--	--
Charaxes castor	--	3	3	--	1	1	--	--	--
Charaxes cedreatis	--	--	--	--	27	27	--	28	28
Charaxes cynthia	52	2	54	18	45	63	20	42	62
Charaxes etesipe	2	--	2	--	1	1	--	1	1
Charaxes etheocles	13	33	46	3	64	67	2	72	74
Charaxes eudoxus	--	--	--	--	1	1	--	--	--
Charaxes eupale	--	--	--	--	30	30	--	38	38
Charaxes fulvescens	1387	18	1405	7	1	8	5	--	5
Charaxes hildabrandti	--	--	--	--	--	--	--	1	1
Charaxes lucretius	--	--	--	3	65	68	4	9	13
Charaxes lycurgus	--	--	--	--	2	2	--	1	1
Charaxes mycerina	--	--	--	--	1	1	--	2	2
Charaxes numenes	42	6	48	2	11	13	1	11	12
Charaxes paphianus	3	1	4	2	13	15	--	9	9
Charaxes petersi	--	--	--	--	3	3	--	--	--
Charaxes plantroui	--	--	--	--	1	1	--	--	--
Charaxes pleione	22	17	39	1	22	23	--	8	8
Charaxes pollux	63	8	71	--	--	--	--	--	--
Charaxes porthos	--	1	1	--	2	2	--	--	--
Charaxes protoclea	29	2	31	18	23	41	32	18	50
Charaxes smaragdalis	2	1	3	--	--	--	--	--	--
Charaxes tiridates	15	19	34	5	42	47	2	22	24
Charaxes varanes	--	--	--	--	--	--	2	--	2
Charaxes viola	--	--	--	--	3	3	--	3	3
Charaxes virilis	--	--	--	--	3	3	--	--	--
Charaxes zelica	1	2	3	--	3	3	--	1	1
Charaxes zingha	--	--	--	3	6	9	--	2	2
Charaxes zoolina	--	4	4	--	--	--	--	--	--
Euxanthe crossleyi	9	6	15	--	--	--	--	--	--
Euxanthe eurinome	--	--	--	--	1	1	1	2	3
Palla decius	--	--	--	4	10	14	4	1	5

Subfamily<br>Species	Kibale National Park<br>Under.	 Canopy	 Abund[a]	Bia Biosphere Reserve<br>Under.	 Canopy	 Abund[a]	Bobiri Forest Reserve<br>Under.	 Canopy	 Abund[a]
Palla publius	--	--	--	1	2	3	2	8	10
Palla ussheri	--	--	--	8	6	14	11	1	12
Palla voilinitens	--	--	--	--	4	4	2	2	4
Heliconiinae									
Lachnoptera anticlia	109	60	169	--	--	--	1	--	1
Phalanta eurytis	26	7	33	--	--	--	--	--	--
Phalanta phalantha	30	171	201	--	--	--	--	--	--
Libytheinae									
Libythea labdaca	--	--	--	--	3	3	--	14	14
Limenitidiane									
Aterica galene	344	6	350	42	--	42	51	--	51
Bebearia abesa	--	--	--	2	--	2	3	--	3
Bebearia absolon	28	--	28	26	--	26	35	--	35
Bebearia arcadius	--	--	--	7	--	7	--	--	--
Bebearia barce	--	--	--	1	--	1	--	--	--
Bebearia carshena	--	--	--	1	--	1	--	--	--
Bebearia cocalia	--	--	--	25	--	25	8	--	8
Bebearia demetra	--	--	--	6	--	6	2	--	2
Bebearia laetitia	--	--	--	1	--	1	--	--	--
Bebearia lucayensis	--	--	--	2	--	2	6	--	6
Bebearia maledicta	--	--	--	1	--	1	--	--	--
Bebearia mandinga	--	--	--	6	--	6	6	--	6
Bebearia mardania	--	--	--	24	--	24	13	--	13
Bebearia oxione	--	--	--	2	--	2	2	--	2
Bebearia paludicola	--	--	--	17	--	17	7	--	7
Bebearia phantasina	--	--	--	24	--	24	21	--	21
Bebearia sophus	249	2	251	81	--	81	29	--	29
Bebearia tentyris	--	--	--	19	--	19	78	--	78
Bebearia zonara	--	--	--	11	--	11	56	--	56
Catuna angustatum	--	--	--	2	--	2	--	--	--
Catuna crithea	207	7	214	2	--	2	2	--	2
Catuna oberthueri	--	--	--	1	--	1	--	--	--
Cymothoe caenias	1	2	3	--	4	4	4	9	13
Cymothoe coccinata	--	--	--	--	1	1	--	--	--
Cymothoe egesta	--	--	--	20	--	20	25	--	25
Cymothoe fumana	--	--	--	3	--	3	--	--	--
Cymothoe herminia	525	90	615	--	--	--	--	--	--
Cymothoe hobarti	92	52	144	--	--	--	--	--	--
Cymothoe jodutta	--	--	--	1	--	1	--	--	--
Cymothoe lurida	416	43	459	--	--	--	2	1	3
Cymothoe mabillei	--	--	--	6	8	14	19	13	32
Cymothoe sangaris	--	--	--	--	--	--	3	--	3
Cymothoe spp.A	--	--	--	--	--	--	1	--	1
Euphaedra alacris	1,344	9	1,353	--	--	--	--	--	--
Euphaedra B217	--	--	--	1	--	1	--	--	--
Euphaedra B301	--	--	--	1	--	1	--	--	--
Euphaedra ceres	--	--	--	89	--	89	86	--	86
Euphaedra cf tenebrosa	--	--	--	1	--	1	--	--	--
Euphaedra christyi	301	5	306	--	--	--	--	--	--
Euphaedra crockeri	--	--	--	41	--	41	--	--	--
Euphaedra diffusa	--	--	--	4	--	4	--	--	--
Euphaedra edwardsii	165	3	168	1	--	1	--	--	--
Euphaedra eleus	--	--	--	3	--	3	--	--	--

Subfamily<br>Species	Kibale National Park			Bia Biosphere Reserve			Bobiri Forest Reserve		
	Under.	Canopy	Abund[a]	Under.	Canopy	Abund[a]	Under.	Canopy	Abund[a]
Euphaedra eupalus	--	--	--	2	--	2	11	--	11
Euphaedra eusemoides	240	--	240	--	--	--	--	--	--
Euphaedra francina	--	--	--	1	--	1	--	--	--
Euphaedra gausape	--	--	--	6	--	6	2	--	2
Euphaedra harpalyce	647	7	654	78	--	78	26	--	26
Euphaedra hebes	--	--	--	7	--	7	4	--	4
Euphaedra hollandi	18	1	19	--	--	--	--	--	--
Euphaedra ignota	--	--	--	3	--	3	--	--	--
Euphaedra janetta	--	--	--	8	--	8	4	--	4
Euphaedra kakamega	35	1	36	--	--	--	--	--	--
Euphaedra mariachristinae	--	--	--	4	--	4	--	--	--
Euphaedra medon	1725	19	1744	32	--	32	100	--	100
Euphaedra minuta	--	--	--	3	--	3	3	--	3
Euphaedra modesta	--	--	--	4	--	4	--	--	--
Euphaedra perseis	--	--	--	9	--	9	--	--	--
Euphaedra phaethusa	--	--	--	61	--	61	48	--	48
Euphaedra preussi	269	5	274	--	--	--	--	--	--
Euphaedra sarcoptera	--	--	--	3	--	3	5	--	5
Euphaedra splendens	--	--	--	2	--	2	1	--	1
Euphaedra themis	--	--	--	3	--	3	43	--	43
Euphaedra uganda	339	3	342	--	--	--	--	--	--
Euphaedra xypete	--	--	--	10	--	10	3	--	3
Euphaedra zaddachi	154	3	157	--	--	--	--	--	--
Euphaedra zampa	--	--	--	5	--	5	--	--	--
Euriphene amicia	--	--	--	1	--	1	1	--	1
Euriphene ampedusa	--	--	--	7	--	7	--	--	--
Euriphene aridatha	--	--	--	13	--	13	26	--	26
Euriphene atossa	--	--	--	15	--	15	2	--	2
Euriphene barombina	--	--	--	38	--	38	70	--	70
Euriphene gambiae	--	--	--	151	--	151	21	--	21
Euriphene incerta	--	--	--	3	--	3	1	--	1
Euriphene lucayensis	--	--	--	--	--	--	2	--	2
Euriphene ribensis	69	2	71	--	--	--	--	--	--
Euriphene saphirina	7	--	7	--	--	--	--	--	--
Euriphene simplex	--	--	--	33	--	33	37	--	37
Euryphura chalcis	4	7	11	6	40	46	6	27	33
Harma theobene	570	23	593	6	--	6	15	--	15
Neptis paula	--	--	--	--	1	1	--	--	--
Neptis strigata	--	--	--	--	1	1	--	--	--
Pseudacraea clarckii	--	1	1	--	--	--	--	--	--
Pseudacraea eurytus	1	--	1	1	--	1	1	1	2
Pseudacraea lucretia	70	107	177	--	--	--	6	4	10
Pseudacraea semire	1	--	1	--	--	--	--	--	--
Pseudathyma falcata	--	--	--	1	10	11	--	--	--
Pseudathyma sibyllina	--	--	--	--	1	1	--	1	1
Pseudoneptis bungandenis	--	--	--	--	--	--	1	--	1
Nymphalinae									
Antanartia delius	40	84	124	--	2	2	--	1	1
Antarnartia dimorphica	11	11	22	--	--	--	--	--	--
Hypolimnas anthedon	3	1	4	--	1	1	--	--	--
Hypolimnas monteironis	2	--	2	--	--	--	--	--	--
Hypolimnas salmacis	5	--	5	2	--	2	3	--	3
Junonia stygia	58	11	69	--	--	--	--	--	--
Junonia terea	--	--	--	--	--	--	1	--	1

Subfamily<br>Species	Kibale National Park			Bia Biosphere Reserve			Bobiri Forest Reserve		
	Under.	Canopy	Abund[a]	Under.	Canopy	Abund[a]	Under.	Canopy	Abund[a]
Junonia westermanni	12	5	17	--	--	--	--	--	--
Kallimoides rumia	369	5	374	--	--	--	5	--	5
Kamilla ansorgei	2	--	2	--	--	--	--	--	--
Protogoniomorpha parhassus	6	2	8	--	--	--	--	--	--
Protogoniomorpha temora	3	--	3	--	--	--	--	--	--
Salamis cacta	58	4	62	4	--	4	--	--	--
Satyrinae									
Bicyclus abnormis	--	--	--	117	10	127	190	36	226
Bicyclus auricruda	576	37	613	8	--	8	2	--	2
Bicyclus buea	350	12	362	--	--	--	--	--	--
Bicyclus campinus	16	--	16	--	--	--	--	--	--
Bicyclus campus	4	--	4	--	--	--	--	--	--
Bicyclus dentatus	74	4	78	--	--	--	--	--	--
Bicyclus dorothea	--	--	--	64	2	66	19	--	19
Bicyclus ephorus	--	--	--	8	--	8	--	--	--
Bicyclus evadne	--	--	--	10	--	10	1	--	1
Bicyclus funebris	18	--	18	153	1	154	1,387	16	1,403
Bicyclus golo	700	16	716	--	--	--	--	--	--
Bicyclus graueri	1,067	8	1,075	--	--	--	--	--	--
Bicyclus ignobilis	--	--	--	2	--	2	--	--	--
Bicyclus istaris	59	--	59	3	--	3	6	3	9
Bicyclus madetes	--	--	--	23	--	23	69	--	69
Bicyclus maesseni	--	--	--	2	2	4	--	--	--
Bicyclus mandanes	826	33	859	5	--	5	--	--	--
Bicyclus martius	--	--	--	16	--	16	68	--	68
Bicyclus mesogena	368	8	376	--	--	--	--	--	--
Bicyclus mollitia	1,790	415	2,205	--	--	--	--	--	--
Bicyclus nobilis	--	--	--	3	--	3	--	--	--
Bicyclus procora	--	--	--	29	--	29	34	--	34
Bicyclus safitza	4	--	4	6	--	6	43	3	46
Bicyclus sambulos	45	1	46	61	--	61	--	--	--
Bicyclus sandace	--	--	--	126	1	127	433	10	443
Bicyclus sangmelinae	--	--	--	68	1	69	25	--	25
Bicyclus sebetus	122	1	123	--	--	--	--	--	--
Bicyclus smithi	9,524	135	9,659	--	--	--	--	--	--
Bicyclus taenias	--	--	--	44	--	44	29	--	29
Bicyclus uniformis	--	--	--	--	--	--	2	--	2
Bicyclus vulgaris	1	--	1	31	1	32	192	1	193
Bicyclus xeneas	--	--	--	24	3	27	63	5	68
Bicyclus zinebi	--	--	--	115	--	115	8	2	10
Elymniopsis bammakoo	--	--	--	4	2	6	11	13	24
Gnophodes betsimena	103	--	103	84	15	99	357	23	380
Gnophodes chelys	955	7	962	41	--	41	52	10	62
Gnophodes grogani	97	4	101	--	--	--	--	--	--
Hallelesis halyma	--	--	--	21	--	21	--	--	--
Henotesia peitho	32	4	36	--	--	--	--	--	--
Melanitis ansorgei	4	--	4	--	--	--	--	--	--
Melanitis leda	96	3	99	44	10	54	156	27	183
Melanitis libya	--	--	--	--	--	--	3	4	7
**Overall**									
Abundance	**27,960**	**4,350**	**32,310**	**2,187**	**577**	**2,764**	**4,151**	**631**	**4,782**
Observed richness	**90**	**75**	**94**	**109**	**59**	**139**	**90**	**54**	**111**
